# Acute safety, cardiovascular, perceptual and neuromuscular responses to autoregulated and non-autoregulated blood flow restriction training during elbow rehabilitation in people with hemophilia

**DOI:** 10.3389/fspor.2025.1587615

**Published:** 2025-07-31

**Authors:** Daniel C. Ogrezeanu, Andrea Tur-Boned, Nicholas Rolnick, Juan J. Carrasco, Carlos Cruz-Montecinos, Joaquín Calatayud, Santiago Bonanad, Sofía Pérez-Alenda

**Affiliations:** ^1^ Exercise Intervention for Health Research Group (EXINH-RG), Department of Physiotherapy, University of Valencia, Valencia, Spain; ^2^ Department of Physiotherapy, University of Valencia, Valencia, Spain; ^3^ The Human Performance Mechanic, New York, NY, United States; ^4^ Department of Exercise Sciences and Recreation, Lehman College, Bronx, NY, United States; ^5^ Physiotherapy in Motion Multispeciality Research Group (PTinMOTION), Department of Physiotherapy, University of Valencia, Valencia, Spain; ^6^ Intelligent Data Analysis Laboratory, University of Valencia, Valencia, Spain; ^7^ Laboratory of Clinical Biomechanics, Department of Physical Therapy, Faculty of Medicine, University of Chile, Santiago, Chile; ^8^ Haemostasis and Thrombosis Unit, Universitary and Polytechnic Hospital La Fe, Valencia, Spain

**Keywords:** electromyography, resistance training, ischemic preconditioning, hemarthrosis, hemophilia A

## Abstract

**Introduction:**

Low-load resistance training with concurrent blood flow restriction (BFR) provides strength and hypertrophy benefits to healthy individuals and some clinical populations. This is the first study assessing safety and physiological responses of autoregulated (AUTO) and non-autoregulated (NAUTO) BFR protocols in people with hemophilia (PWH). Therefore, this study aimed to evaluate the acute safety, cardiovascular, neuromuscular and perceptual responses during AUTO and NAUTO BFR training in PWH.

**Methods:**

Eleven severe PWH under prophylaxis performed two sessions of elbow flexion and extension using elastic bands at 50% of the limb occlusion pressure (LOP) with different BFR settings (AUTO vs. NAUTO). Safety, cardiovascular parameters, rating of perceived exertion, elbow pain and pressure algometry were assessed at different timepoints. High-density surface electromyography activity and its spatial distribution were determined for biceps and triceps brachii.

**Results:**

Both BFR conditions were safe in PWH. AUTO provided a hypotensive and hypoalgesic acute response, albeit without between-group differences. Triceps brachii showed differences in spatial distribution, and greater activity with AUTO in the last 3 cycles of the first 3 sets. Although no major differences were found between both conditions in perceptual responses, AUTO condition increased VAS scores during both exercises. No adverse events were observed.

**Conclusions:**

BFR at 50%LOP during arm exercise with either AUTO or NAUTO appears to be equally safe in PWH, but AUTO showed trends for improved cardiovascular and neuromuscular responses. AUTO produced a hypotensive and hypoalgesic acute post-exercise response, albeit without between-group differences, a greater activation in triceps brachii, and higher values of pain. No serious adverse events were observed.

## 1 Introduction

Hemophilia is an X-linked congenital bleeding disorder caused by a deficiency of the clotting factors VIII or IX (1). A bleeding tendency is characteristic in severe cases, most commonly in synovial joints like the elbows (1). A single bleed or recurrent bleeds may cause hemophilic arthropathy, causing joint destruction with irreversible changes in the cartilage and bone tissue, leading patients to experience pain and limitation in daily activities (2).

Exercise programmes are usually designed and implemented to help manage the recovery after a bleed or to prevent bleeding episodes (3). To improve muscle strength, regular heavy-load resistance training with external loads of 60%–90% of one-repetition maximum (1RM) is recommended (4). However, some studies have demonstrated similar gains in strength (5) and hypertrophy (6) between high-load training and low-load training (20%–30% 1RM) with blood flow restriction (BFR). BFR training (BFRT) consists in applying an external pressure to the most proximal region of the limb, causing a full restriction of venous outflow while maintaining arterial inflow of blood distal to the cuff (7). This is thought to induce a hypoxic environment leading to increased levels of metabolic stress, a rise in type II muscle fibre recruitment, and the accumulation of metabolites (8). BFRT may be appropriate in patients who may not be able to tolerate heavy-loads (9) such as people with hemophilia (PWH), where a nuanced equilibrium between improving strength and risk of joint injury exists (3). This complex equilibrium impacts prescription of exercise regimens. In addition, BFRT enables shorter training sessions, which, together with the low intensity used, could help manage kinesiophobia and catastrophism that is usually present in PWH (10).

To reduce the potential for excessive stress on the cardiovascular system (e.g., blood pressure increase), the use of personalized limb occlusion pressures (LOP) is recommended (7). Nevertheless, LOP is statically determined at rest and does not account for muscle contractions. Previous studies have shown an increase in intramuscular pressure with reduced blood flow during contractions (11, 12). This happens with non-autoregulated (NAUTO) BFR devices in which the pressure applied to the limb is not adapted to the phase of muscular contraction, leading to variable levels of vascular occlusion (13, 14). Conversely, autoregulated (AUTO) devices avoid variability in applied pressure to the limb by monitoring for pressure changes and adjusting pressure dynamically, partially deflating when surpassing the target percentage of LOP and reinflating when pressure goes below the desired level, rapidly compensating the higher than desired occlusion levels that occur during muscle contractions (14). Theoretically, AUTO should be better tolerated than NAUTO, resulting in improved clinical outcomes. However, only a few number of studies (15, 16) thus far have examined the acute differences between AUTO and NAUTO, all conducted in healthy people and reporting no major differences in cardiovascular outcomes (e.g., brachial blood pressures). In one study (15), AUTO appeared to reduce the risk for adverse events, muscle soreness after 24 h and was perceived to be significantly less uncomfortable; however, the other study found no differences in perceptual experiences or differences in mitigating adverse responses as both conditions did not induce any adverse events (16). In addition, no previous studies have investigated the neuromuscular differences between NAUTO and AUTO in clinical population, which could provide useful information for clinical decision-making. This underscores the necessity for further studies to be conducted wherein BFR pressure application settings are applied among populations with musculoskeletal conditions that stand to derive the greatest benefit from them. Thus far, only two studies have implemented BFR resistance training in PWH (17, 18). One demonstrated safety, albeit within a small number of repetitions (17), while the other implemented a full standard BFR session (18). However, both only used a traditional NAUTO device and only during one exercise in the lower body (17, 18). Hence, the purpose of this study was to examine the acute safety, cardiovascular, neuromuscular and perceptual responses during AUTO and NAUTO BFRT in the upper body in PWH.

## 2 Methods

### 2.1 Participants

Candidates were adult PWH A or B (moderate or severe), 18–60 years old and undergoing prophylaxis. Participants were excluded if they (1) had surgical procedures performed 6 months prior to the exercise program; (2) participated in any other form of exercise, not previously done, during the study; (3) had any changes in medication during the study; (4) had a major bleeding episode that posed a risk or prevented exercise 6 weeks prior to or during the study; (5) had another hemostatic defect; (6) had history of stroke, brain surgery, major depression, or any self-perceived cognitive alterations that could affect the performance of study tasks. Participants were duly informed and gave written informed consent. The study conformed to the Declaration of Helsinki, was approved by the local Ethics Committee, and adheres to STROBE guidelines.

### 2.2 Procedures

Demographics and clinical data were collected from recent medical records and interviews. Participants were asked to not consume any nourishment, alcohol, or stimulants in the 2 h prior to the sessions. They were asked to not engage in any form of physical activity more intense than basic activities in the 24 h before the sessions nor use any analgesics, and they were also recommended to sleep a minimum of 7–8 h the night before.

Participants attended two sessions separated by approximately one week (mean 9.6 days; SD 4.1). Each session, an intervention was assigned in a counterbalanced manner, including elbow extensions and flexions, speed and external resistance matched, but with a different BFR protocol: (1) occlusion at 50% LOP with non-autoregulated pressure setting (NAUTO); (2) occlusion at 50% LOP with autoregulated pressure setting (AUTO). On the first day, the following baseline assessments were collected: elbow pain intensity was assessed using the 100 mm visual analogue scale (VAS) while kinesiophobia was assessed using the Tampa Scale for Kinesiophobia (TSK-11). Leisure-time physical activity and resistance training experience were also assessed.

Then, with the participants seated, measures of cardiovascular stress [systolic blood pressure (SBP), diastolic blood pressure (DBP) and heart rate (HR)] were evaluated in the dominant arm using an automatic tensiometer (M3 Comfort, OMRON Healthcare, Japan). Three measurements were taken (separated by 2 min) and averaged at each timepoint: preexercise (pre), immediate postexercise (post), and 10 min after exercise (post 10 min). Also, the mean arterial pressure (MAP), taking into account DBP, HR and pulse pressure (PP; which is the difference between SBP and DBP), was calculated with the formula: MAP = DBP + [0.01 × EXP(4.14–40.74/HR) × (PP)]. Afterwards, pressure pain thresholds (PPT) were evaluated using a digital pressure algometer (NOD, OT Bioelettronica, Italy), 3 cm distal to the dominant arm lateral epicondyle, in the extensor digitorum muscle tendon. Three PPT measurements were taken (separated by 30 s) and averaged at each timepoint.

Subsequently, as a baseline safety precaution measure, an ultrasound scan (LOGIQ C5 Premium, GE Healthcare, USA) of the exercising arm (the dominant one) was performed to exclude subclinical active bleeding in the biceps and triceps brachii. Next, active elbow extension/flexion range of motion (ROM) was measured using a goniometer, with the participants standing. Three measurements were taken for each movement and their mean registered.

Exercise intensity was identified using elastic bands (TheraBand CLX, Performance Health, USA) progressively from lowest to highest resistance (yellow color to red, green, blue, black, silver or gold). Participants performed 2–3 sets of 2 reps with 60 s rests until they rated a 2 on Borg's CR10 Scale (corresponds to 30% 1RM) (19). A 5 min rest was then taken during which, with the participants standing, the high-density surface electromyography (HDsEMG) protocol began with skin marking in the biceps brachii and triceps brachii muscles (20). After the skin was shaved and abraded to remove dead skin cells and cleaned with cotton wool dipped in alcohol, the electrode grids were positioned, with the electrode columns oriented along the muscle fibers. The reference electrodes were placed at the wrist. Specifically, HDsEMG was recorded in monopolar derivation with semi-disposable adhesive matrices (GR10MM0804, OT Bioelettronica, Italy) of 32 (8 × 4) equally spaced electrodes (with an inter-electrode distance of 10 mm). HDsEMG signals were sampled at 2,000 Hz and converted to digital data by a 16-bit analogue to digital converter (Sessantaquattro, 64-channel HDsEMG, OT Bioelettronica, Italy).

Before the training and after a submaximal practice trial, participants performed two maximum voluntary isometric contractions (MVIC) for each muscle (with 30 s rest) to normalize HDsEMG (to the highest MVIC or the highest in session amplitude, if higher), and to measure maximum isometric elbow flexion and extension strength by performing the MVICs against a fixed hand-held dynamometer (NOD, OT Bioelettronica, Italy). Participants sat with erect posture and no back support at 90° of elbow flexion. In this position, participants performed a 2 s progressive ramp contraction and then maintained a maximum contraction effort for the next 3 s. Participants were verbally encouraged to reach their maximal effort.

After a 5 min rest, the LOP of the exercising upper limb was determined with the participants standing while the pneumatic cuff (SmartCuffs PRO 3, Smart Tools Plus, USA) was placed on the most proximal portion of their arm (cuff width, 6.35 cm). The inflation procedure was automated and based on an in-device algorithm validated against the current Doppler ultrasound gold standard (21). Once LOP was determined, the cuff was deflated, and the participants rested quietly for 5 min before training. For safety, a pulse oximeter (CMS50D, Contec Medical Systems Co., China) was used on the thumb throughout the session.

The exercise protocol followed the standard BFRT structure of four sets (30, 15, 15, 15 reps; 30 s rests) with continuous BFR (5 min break between exercises for reperfusion). The elastic bands were pre-stretched (adding about 25% of the initial length) and a metronome was used to ensure a cadence of 1.5 s per movement phase. After performing each set, HR and oxygen saturation (SaO2) were assessed with the pulse oximeter and participants were asked to rate their rating of perceived exertion (RPE) on the Borg CR10 scale, their pain intensity in the exercising arm with the VAS, and degree of perceived tolerability using a five-point scale (i.e., very well tolerated = 5, tolerated = 4, neutral = 3, not well tolerated = 2 and not tolerated = 1). Moreover, after finishing the two exercises, SBP, DBP, HR, VAS and PPTs were reassessed immediately and at 10 min after. Subsequently, participants completed a global change scale about the potential change in their fear of practicing BFRT (very much improved, much improved, minimally improved, no change, minimally worse, much worse, very much worse). Finally, 24, 48 and 72 h after each session, participants were interviewed about delayed onset muscle soreness (DOMS), joint pain, and stiffness using an 11-point scale after palpating the arm and moving from full flexion to full extension. They were also asked about any suspicion of muscle or joint bleeds, or any possible adverse effects, and instructed to report any they might feel during the week after.

### 2.3 HDsEMG data analysis

The HDsEMG signals were processed offline using custom-made algorithms implemented in MATLAB software (The MathWorks Inc., Natick, Massachusetts, USA, version R2018b). All raw signals were amplified to obtain the EMG data in microvolts. Next, a differentiation of the 32-monopolar HDsEMG channels was carried out along the fibers direction (columns of the grid) to obtain arrays of 7 columns × 4 rows of bipolar signals, i.e., 28 RMS signals on each electrode array. A Butterworth fourth-order zero-lag band-pass filter (20–400 Hz) was then applied to each signal to eliminate low and high-frequency noise. Subsequently, a visual inspection was carried out to discard signals with excess noise. A moving root-mean-squared (RMS) smoothing filter was applied to the HDsEMG signals, implemented with a 1,000 ms window (500 ms backward and 500 ms forward) for each signal sample.

Once the signals were filtered, an automatic segmentation of the contractions was carried out from the maximum and minimum peaks of each signal. This methodology allows us to delimit the period of muscle activity, but not to distinguish the concentric and eccentric phases of muscle contractions. However, it has been used before in many previously published studies (17, 18, 22). While it is true that contraction mode can affect EMG amplitude, and many studies synchronize EMG with other types of additional external signals to be able to do this (video recording, motion capture, and accelerometer data) (23, 24), we chose to analyze the exercise sets with no differentiation between the concentric and eccentric components, as patients normally complete both components together during their rehabilitation programmes.

In each of the contractions, the mean RMS activation percentage (amplitude) was obtained by normalizing the result to the highest RMS activation value reached by the participant during the session (including MVIC). After obtaining these normalized variables in each signal of the map (7 × 4 matrix signals), the average nRMS values were obtained, as well as the coordinates of the HDsEMG nRMS map centroid (x- and *y*-axis coordinates for the medial-lateral and cranial-caudal direction, respectively) and the modified entropy. The average nRMS HDsEMG from all channels on the matrix was used as a parameter of muscle activation, while the displacement of the centroid and variations in the modified entropy were used to assess HDsEMG activity spatial distribution. A higher modified entropy (from a maximum possible value of 4.81 in the case of our matrices of 28 RMS signals) represents less heterogeneity in the spatial distribution of nRMS values within the electrode matrix, ergo higher homogeneity, whilst a decrease in entropy indicates a decrease of homogeneity. Coefficient of variation (CoV) was defined as the standard deviation (SD) of the 28 RMS values divided by the average of the 28 RMS values. When SD is small relative to the mean, this results in a smaller CoV. Therefore, when channel signals are more uniform, there will be a smaller CoV to also indicate increased homogeneity (reduced heterogeneity). To allow statistical analysis, the repetitions of each set were averaged in successive cycles. In the case of set 1 (30 reps), each cycle consists of the average of 6 reps. In sets 2–4 (15 reps), each cycle is the result of averaging 3 reps. In all cases, 5 cycles were obtained.

### 2.4 Statistical analysis

An *a priori* power analysis was conducted (G∗Power; Düsseldorf, Germany) to calculate the required sample size. With the present study design, assuming a medium effect size (f = 0.30), a 5% alpha risk (*α* = 0.05) and 20% beta risk (*β* = 0.2; powe*r* = 0.80), and a correlation between repeated measures of 0.6, a total of 11 participants were sufficient.

The statistical analysis was performed with SPSS v26 (IBM Corp, USA). The normality of the data was verified with the Shapiro–Wilk test. Descriptive results are shown as mean (SD), median [25th−75th percentiles] or n (percentages) as appropriate.

The differences between conditions (AUTO, NAUTO) and times (pre, post, post 10 min) for SBP, DBP, HR, MAP, VAS and PPT were evaluated using linear mixed models. The conditions and times were entered into the model as repeated measures with fixed effects.

RPE, VAS, tolerability and HR were evaluated using linear mixed models to analyze differences between conditions and sets (1–4). The conditions and sets were configured as repeated measures with fixed effects.

The differences between conditions, sets and cycles (1–5) for nRMS, CoV, modified entropy and centroid were evaluated using linear mixed models. The conditions, sets and cycles were considered as repeated measures with fixed effects. In all mixed models, subject was entered as random effect and the restricted maximum likelihood estimation method was used with the Satterwait approximation. When the main effects indicated significant differences, the Bonferroni correction was applied to avoid Type I error caused by multiple comparisons. Data were statistically significant when *p* < 0.05.

## 3 Results

### 3.1 Participants

Eleven adults with hemophilia A participated in the study. Demographic and clinical data are shown in Table 1. About half (54.55%) of the participants had a history of resistance training and were involved in physical activity for at least 1 day·week^–1^. No serious adverse effects were reported during the sessions, with none of the reported events impeding the completion of the exercises.

**Table 1:** Demographic and clinical data.

(*n* = 11)	Mean (SD) or median [25th–75th percentile]
Age (years)	39.4 (11.9)
Height (cm)	175.5 (8.8)
Body mass (kg)	80.0 (9.4)
FVIII dose (IU/Kg)	27.7 (10.5)
FVIII dose (IU/week)	4,000.0 (4,000.0–6,000.0)
Hours since last SHL FVIII dose (*n* = 5)	5.43 (8.30)
Hours since last EHL FVIII dose (*n* = 6)	13.50 (29.43)
HJHS dominant elbow	2.0 (0.0–7.0)
HJHS non dominant elbow	3.0 (2.0–10.0)
HJHS total	25.2 (15.2)
Elbow flexion ROM (°)	128.5 (7.6)
Elbow extension ROM (°)	−18.2 (15.6)
Isometric elbow flexion strength (N)	169.5 (38.8)
Isometric elbow extension strength (N)	143.5 (21.3)
VAS score (mm)	8.2 (16.6)
NAUTO LOP (mmHg)	198.2 (30.9)
NAUTO 50% LOP (mmHg)	99.1 (4.7)
AUTO LOP (mmHg)	182.7 (20.5)
AUTO 50% LOP (mmHg)	91.4 (10.3)
TSK-11 (Total: 11–44)	26.2 (3.1)
Leisure-time physical activity	
**Frequency**	***n* (%)**
Never	1 (9.1)
1 time/week	0 (0)
2–3 times/week	7 (63.6)
Almost daily	3 (27.3)
Resistance training experience	
Yes	6 (54.5)
No	5 (45.5)
Frequency	
1 time/week	1 (9.1)
2 times/week	3 (27.3)
3 times/week	2 (18.2)
Years of experience	
<5 years	3 (50.0)
>5 years	3 (50.0)
Fear of practicing BFR training (baseline)	
0	9 (81.8)
3	1 (9.1)
7	1 (9.1)
Fear of practicing BFR training (change)	
Very much improved	1 (9.1)
Much improved	1 (9.1)
No change	9 (81.8)

FVIII, coagulation factor VIII; SHL, standard half-life factor; EHL, extended half-life factor; HJHS, haemophilia joint health score; ROM, range of motion; VAS, visual analogue scale; LOP, limb occlusion pressure; TSK-11, 11-item Tampa Scale for Kinesiophobia; NAUTO, non-autoregulated; AUTO, autoregulated; SD, standard deviation; cm, centimeters; kg, kilograms; IU, international units;°, degrees, N, newtons; mmHg, millimeters of mercury; BFR, blood flow restriction.

### 3.2 Cardiovascular and hypoalgesic responses

The acute cardiovascular and hypoalgesic responses to NAUTO and AUTO BFRT are shown in Table 2. The AUTO condition was the only one that produced a hypotensive and hypoalgesic acute response, albeit there were no between-group differences.

**Table 2:** Acute cardiovascular and hypoalgesic responses before and after the blood flow restriction resistance training session.

		Pre	Post	Post 10 min	Pre vs. Post (p; d)	Pre vs. Post 10 min (p; d)
Systolic blood pressure (mmHg)	NAUTO	128.5 (10.8)	127.6 (9.3)	126.1 (9.0)	–	–
	AUTO	128.9 (10.5)	121.1 (8.2)	120.0 (9.0)	**.011; 0.83**	**.020; 0.91**
Diastolic blood pressure (mmHg)	NAUTO	80.3 (8.7)	76.4 (8.8)	78.9 (7.8)	–	–
	AUTO	80.3 (9.0)	74.4 (7.8)	76.6 (8.2)	**.002; 0.70**	–
Heart rate (bpm)	NAUTO	74.9 (15.9)	73.6 (13.5)	72.3 (13.7)	–	–
	AUTO	81.7 (15.8)*	76.8 (15.5)	75.0 (15.2)	**.041; 0.31**	**.016; 0.44**
Mean arterial pressure (mmHg)	NAUTO	97.6 (10.4)	94.6 (8.9)	95.6 (8.9)	–	–
	AUTO	98.5 (9.3)	91.2 (7.9)	92.0 (8.6)	**<.001; 0.84**	**.013; 0.72**
PPT	NAUTO	347.5 (86.3)	383.2 (68.6)	343.7 (71.7)	–	–
	AUTO	402.3 (168.7)	336.9 (83.9)	322.4 (75.4)	–	**.022; 0.61**
VAS	NAUTO	2.73 (9.05)	3.64 (9.24)	3.64 (9.24)	–	–
	AUTO	8.18 (16.62)	11.36 (20.01)	2.73 (9.05)	–	–

Bold text denotes significant differences while “-” means no significant differences. Comparisons between NAUTO and AUTO marked only when significant (*p = 0.009; *d* = 0.43). p, *p*-value; d, Cohen's d effect size; NAUTO, non-autoregulated; AUTO, autoregulated; PPT, pressure pain thresholds; Pre, pre-exercise; Post, immediate postexercise; Post 10 min, 10 min after exercise, vs., versus; mmHg, millimeters of mercury; bpm, beats per minute.

### 3.3 High-density surface EMG

**Figure 1:**
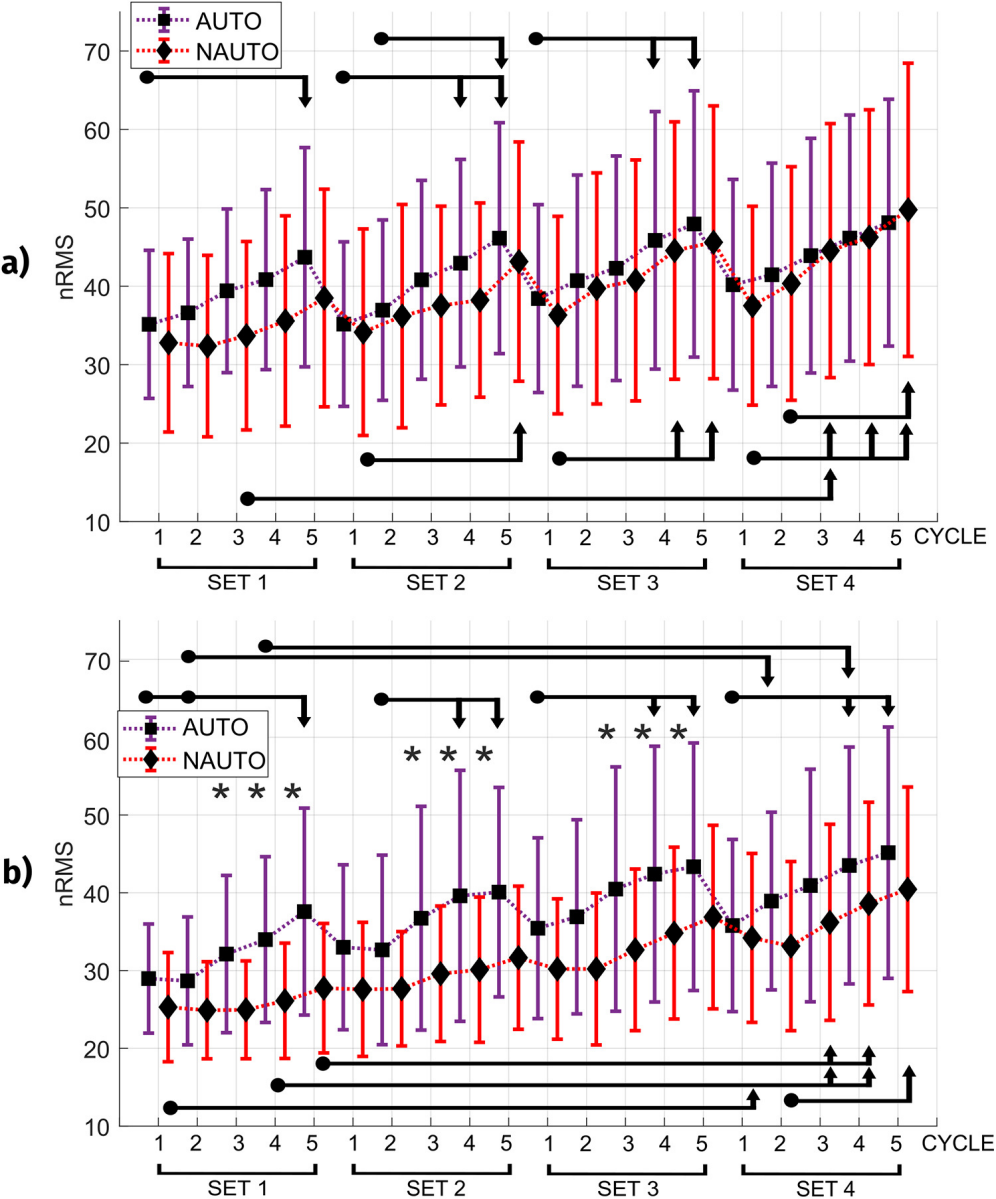
Normalized values of electromyographic amplitude (nRMS) for each condition. **(a)** Biceps brachii **(b)** Triceps brachii. *Indicates a difference between the two blood flow restriction conditions (AUTO and NAUTO). The start and end of the arrow indicate the compared intervals. Those at the top of the figure represent the AUTO condition, while those at the bottom represent the NAUTO condition. AUTO, autoregulated; NAUTO, non-autoregulated; nRMS, normalized root-mean-square.

Neuromuscular activity (mean %nRMS for biceps and triceps brachii muscles) during both BFR conditions is shown in Figure 1. While biceps brachii had no difference between both conditions, triceps brachii showed a greater activation with the AUTO condition in the last 3 cycles of the first 3 sets.

**Figure 2:**
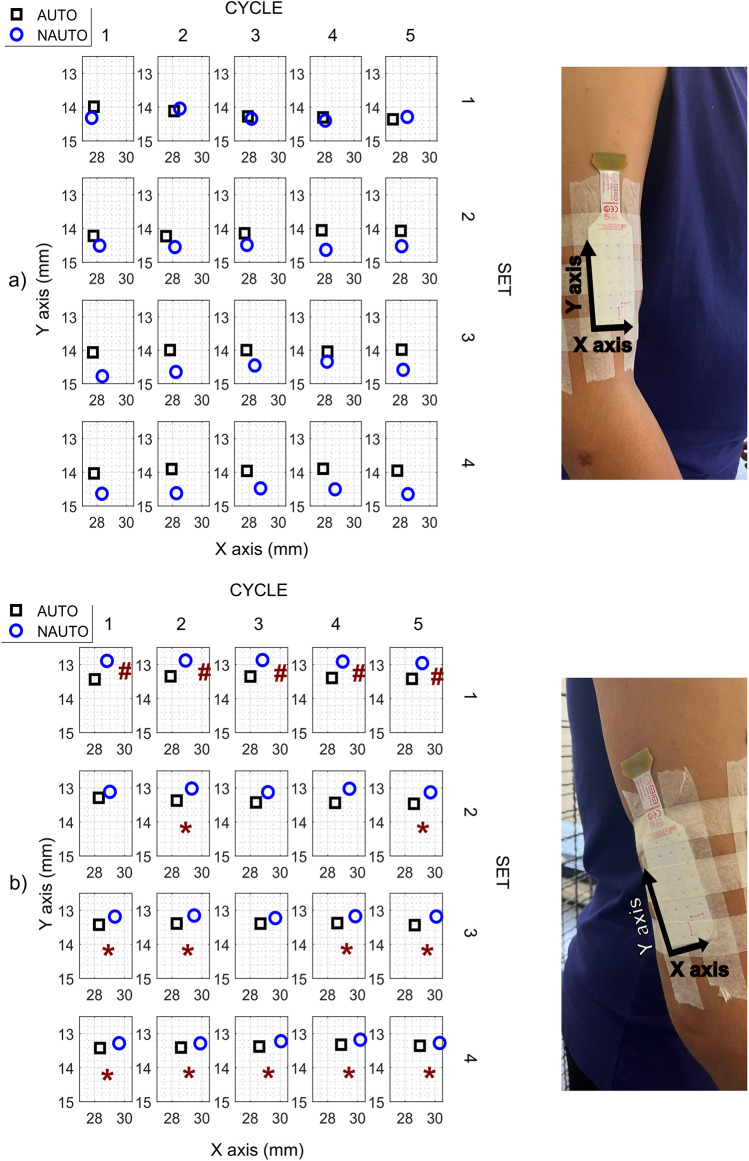
Mean values of normalized values of electromyographic amplitude (nRMS) maps centroid in each condition and placement of the high-density surface electromyography electrodes. **(a)** Biceps brachii **(b)** Triceps brachii. *Indicates differences on the *x*-axis (medial-lateral direction), while the # indicates differences on the *y*-axis (cranial-caudal direction) in each of the 5 cycles of each one of the 4 exercise sets. AUTO, autoregulated; NAUTO, non-autoregulated; mm, millimeters.

The mean locations for the nRMS map centroid in the biceps and triceps brachii obtained in each BFR condition are represented in Figure 2. The biceps brachii showed no statistically significant differences between both pressure settings in spatial distribution. Regarding the displacement of the map centroid in the triceps brachii, differences between both conditions (*p* < 0.05) were found in both axes, with NAUTO exhibiting a cranial migration in *y*-axis and a lateral migration in *x*-axis. In addition, the modified entropy did not change.

### 3.4 Perceptual responses, heart rate and oxygen saturation

The acute perceptual responses, HR and SaO2 after each exercise set of BFRT are shown in Table 3. Overall, there were no major differences between both types of BFR pressure application setting. However, compared to NAUTO, the AUTO condition increased VAS scores during each set of exercise with elbow flexion and during sets 2–4 with elbow extension. Both BFR types were equally tolerable and safe, without great increases in RPE or high pain levels.

**Table 3:** Acute perceptual and cardiovascular responses after each set of blood flow restriction resistance training.

			SET 1	SET 2	SET 3	SET 4
RPE	FLEX	NAUTO	2.6 (1.4)	2.3 (1.1)	2.5 (1.0)*	3.2 (1.4)
		AUTO	2.7 (1.1)	2.7 (1.1)	3.0 (1.5)	3.3 (1.6)
	EXT	NAUTO	2.6 (1.3)*	2.7 (1.1)**	3.5 (1.1)	3.9 (1.4)
		AUTO	2.9 (0.7)*	3.4 (1.0)	3.5 (1.3)	4.1 (1.9)
VAS	FLEX	NAUTO	1.8 (6.0)***	1.8 (6.0)***	2.7 (9.0)***	2.7 (9.0)***
		AUTO	11.4 (16.7)	11.4 (16.7)	12.3 (18.1)	16.8 (20.8)
	EXT	NAUTO	2.7 (9.0)	2.7 (9.0)***	2.7 (9.0)***	3.0 (9.5)***
		AUTO	10.9 (18.7)	13.2 (23.1)	12.3 (22.1)	12.3 (22.1)
TOLERABILITY	FLEX	NAUTO	4.2 (0.6)	4.4 (0.5)	3.9 (0.8)	3.7 (0.8)
		AUTO	3.9 (0.9)	3.9 (0.9)	3.7 (0.9)	3.5 (0.8)
	EXT	NAUTO	4.3 (0.6)	4.1 (0.5)	3.8 (1.0)	3.6 (1.1)
		AUTO	4.1 (0.7)	3.8 (0.9)	3.8 (0.9)	3.8 (0.9)
HR	FLEX	NAUTO	75.5 (25.8)	63.7 (25.6)	62.9 (26.6)	77.4 (17.2)
		AUTO	70.7 (30.4)	74.8 (30.0)	79.0 (24.8)	83.3 (16.2)
	EXT	NAUTO	71.5 (26.4)	60.6 (24.7)	66.6 (28.4)	59.9 (26.3)
		AUTO	80.6 (55.0)	76.5 (28.8)	61.5 (30.6)	73.5 (24.1)
SaO2	FLEX	NAUTO	96.9 (1.3)	96.0 (2.3)	96.3 (2.1)	96.7 (1.3)
		AUTO	97.2 (1.2)	96.8 (2.5)	96.3 (3.1)	96.6 (2.7)
	EXT	NAUTO	96.1 (2.3)***	96.8 (1.8)	96.3 (3.8)	97.8 (1.3)
		AUTO	92.2 (5.8)	94.5 (7.6)	94.2 (7.5)	94.7 (7.4)

* Significant differences compared with SET 4 (*p* < 0.05).

** Significant differences compared with SET 3 and with SET 4 (*p* < 0.05).

*** Significant differences between NAUTO and AUTO (*p* < 0.05). RPE, rating of perceived exertion; FLEX, elbow flexion; EXT, elbow extension; NAUTO, non-autoregulated; AUTO, autoregulated; VAS, visual analogue scale; HR, heart rate; SaO2, oxygen saturation of arterial blood.

### 3.5 Adverse events

To better contextualize the safety profile of BFRT in severe PWH, adverse events as they relate to BFRT and the characteristics of PWH were considered as: (a) minor and/or expected due to BFR application (tightness in arm, itchy hand/forearm, finger congestion); (b) minor and/or expected due to resistance exercise and PWH fitness (DOMS, stiffness); (c) minor and/or expected due to the characteristics of PWH and their arthropathy status (elbow or shoulder crepitation); (d) clinically relevant concerns due to the characteristics of PWH and their arthropathy status (superficial hematoma from BFR cuff; elbow or shoulder pain; elbow or shoulder stiffness; elbow or shoulder joint bleeds; muscle bleeds). Most reports (Table 4) consisted of feeling tightness in the arm (4 NAUTO vs. 3 AUTO), with two reports from the same participant in NAUTO, two more from another in AUTO, and one in each condition from the same participant. Additionally, one participant reported tightness and elbow crepitation (due to arthropathy) in the NAUTO condition, while another one reported finger congestion in the AUTO condition. One participant reported feeling a light shoulder pain during elbow flexion exercise in AUTO. Another participant reported feeling itchiness in the hand and forearm in both conditions. Also, one participant presented a slight superficial hematoma caused by the BFR cuff (NAUTO), and another one unexpectedly suffered an insignificant superficial skin abrasion caused by the sharp edge of the EMG matrix in the elbow crease (NAUTO). Additionally, participants reported their levels of DOMS, joint pain, and stiffness after 24, 48 and 72 h. The most reported were DOMS at 24 and 48 h, especially in AUTO. Only one participant reported a slight exacerbation of his elbow extension joint pain (to a 4 on the VAS, from a baseline level of 2). Another participant reported feeling elbow stiffness (without pain) 24 h post AUTO.

**Table 4:** Adverse events within 11 participants who completed all sessions.

	NAUTO			AUTO		
Number of independent adverse events reported during the sessions.						
Tightness in arm	4			3		
Elbow crepitation	1			0		
Itchy hand/forearm	1			1		
Shoulder pain	0			1		
Finger congestion	0			1		
BFR cuff superficial hematoma	1			0		
Elbow crease skin abrasion (EMG matrix)	1			0		
Adverse effects reported 24, 48 and 72 h after the sessions (Mean ± SD).						
	24 h	48 h	72 h	24 h	48 h	72 h
DOMS Biceps	0.0 (0.0)	0.0 (0.0)	0.0 (0.0)	0.7 (1.3)	0.5 (1.0)	0.1 (0.3)
DOMS Triceps	0.3 (0.9)	0.1 (0.3)	0.0 (0.0)	0.8 (1.5)	0.5 (1.0)	0.2 (0.4)
Joint pain	0.4 (1.2)	0.2 (0.6)	0.2 (0.6)	0.4 (1.2)	0.3 (0.9)	0.2 (0.6)
Stiffness	0.0 (0.0)	0.0 (0.0)	0.0 (0.0)	0.5 (1.8)	0.0 (0.0)	0.0 (0.0)

NAUTO, non-autoregulated; AUTO, autoregulated; BFR, blood flow restriction; EMG, electromyography; SD, standard deviation; DOMS, delayed onset muscle soreness; h, hours.

## 4 Discussion

This is the first study to examine the acute safety, cardiovascular, neuromuscular and perceptual responses during AUTO and NAUTO BFRT in PWH. The main and novel findings were: (1) the AUTO condition was the only one providing a hypotensive and hypoalgesic acute response, albeit no between-group differences were found; (2) the AUTO condition induced greater triceps brachii activation in the last 3 cycles of the first 3 sets; (3) compared to NAUTO, the AUTO condition showed higher VAS scores during each set of elbow flexion exercise and during the last 3 sets of elbow extension; (4) both BFR types were equally tolerable and safe, without great increases in RPE or high pain levels; (5) no serious adverse events were reported during the experimental sessions and during the first week.

Our results support the idea that exercise can provide an acute post-exercise hypotensive response. However, this result was only evident after the AUTO condition, albeit no differences were found between conditions. A recent meta-analysis demonstrated a decrease in DBP when applying intermittent BFR with low-load upper limb training in healthy people (25). Regrettably, no previous studies evaluate pre and post exercise cardiovascular outcomes with different BFR types. Furthermore, Jacobs et al. (15) found an increase of cardiovascular outcomes (SBP, DBP, HR, MAP) during a fixed protocol with both BFR conditions in healthy individuals, albeit differences between AUTO and NAUTO were not significant. These higher cardiovascular results while training could be explained by an increase in sympathetic nervous system activity maintained and augmented via feedback from baroreceptors located in the aorta and carotid artery, as well as by stimulation of mechanically and metabolically sensitive receptors in skeletal muscle (26). Then, the acute hypotensive response produced after exercise could be explained by a reactivation of parasympathetic nervous system (26) and by reactive hyperemia (vasodilator substances increase) following cuff removal (27).

Our nRMS results show no differences between both BFR conditions in biceps brachii, but a greater activation of triceps brachii with the AUTO condition in the last 3 cycles of the first 3 sets. Partly in line, Bordessa et al. (28) found no significant differences between both BFR conditions in peak or average quadriceps EMG activity, albeit among healthy people and using different devices. Concerning the muscle activity increase, two studies (17, 28) demonstrated differences in neuromuscular activation depending on the external load, not on the cuff occlusion. However, both biceps and triceps brachii muscle activation increased progressively across the four sets conducted in our study, in accordance with previous findings evaluating a BFRT session with low-load elastic resistance (29). Unfortunately, no previous studies were conducted on PWH comparing both BFR types. We also observed shifts in the nRMS map of the triceps brachii, specifically the cranial and lateral migrations in the NAUTO condition. These shifts suggest acute adaptive responses in redistribution of activity similar to effects seen in endurance tasks in other muscle groups, such as the trapezius (30) and back muscles (31). It is thought that this redistribution of activity likely prevents localized muscle fatigue (32). However, there were no differences in entropy, indicating that the overall homogeneity in the spatial distribution of nRMS values within the electrode matrix was similar between conditions.

Concerning the acute perceptual responses, there were no major differences between both types of BFR pressure application. However, in general, we found higher RPE values during elbow extension than during elbow flexion and higher RPE during the last set, concurring with our nRMS results. A previous study performed in healthy individuals, found higher values during leg extension training with AUTO than with NAUTO (28). Furthermore, Jacobs et al. (15) found higher RPE values over time in the NAUTO device among healthy individuals in a leg extension BFR fixed protocol. Conversely, another recent study showed no differences in perceptual responses between NAUTO and AUTO in multi-joint lower body failure exercise using the Delfi device (16). Therefore, the perceptual responses elicited with AUTO/NAUTO may be device specific, region specific (e.g., upper versus lower limb), or protocol specific (e.g., fixed versus failure) and likely requires some consideration during the implementation process. Partly in line with our VAS results, Bordessa et al. (28) found higher values of pain in the AUTO condition among healthy individuals in a leg extension training protocol. However, concerns were made regarding the comparisons given the cuff design differences between conditions (33). However, our VAS values with both conditions were low, with most participants reporting no pain, which could be explained by a greater tolerance to pain in PWH, potentially due to their habituation to recurrent painful episodes associated with the condition, or by substantial variability in individual pain perception. Nevertheless, both BFR conditions were equally well tolerated. In fact, only 8 adverse events occurred during the NAUTO session, while 6 adverse events appeared during the AUTO session, none of them impeding the completion of the exercises. Some of the reported events happened in the same participants and could be explained due to the restriction of blood flow distal to the cuff location. Slightly in line with these results, Jacobs et al. (15) also found a higher number of adverse events (presyncopal symptoms, numbness in leg and exercise intolerance) during NAUTO training sessions, with presyncopal symptoms as the most common event. However, in our study, the adverse events did not impede participants to complete the session, while in the Jacobs et al. (15) study 10 participants stopped exercising due to presyncopal symptoms during sessions. After all, no bleeding events were reported during our study nor in the first week post sessions, which is the most important finding in terms of safety for PWH. All in all, our findings infer that both types of BFR can be used, potentially informing clinical decision-making in hemophilia care, physiotherapy or exercise prescription contexts. However, specialists should consider using AUTO to increase triceps activation and when cardiovascular responses are relevant to patients. Additionally, the EMG activity spatial distribution variability seen with NAUTO seems to reflect a necessity to compensate a superior accumulation of metabolic stress and peripheral fatigue possibly due to impaired contractile function because of the higher pressures applied to the limb.

Our study is not without limitations. The number of participants was small, although HDsEMG has high reliability, and measurements were conducted among patients with a rare disease. In addition, differentiating concentric and eccentric phases could provide deeper insights into task-specific neuromuscular control. However, in our rehabilitation context, we prioritized the magnitude of the EMG activity. Given the modest sample size and the use of a medium effect size in our power analysis, there remains a risk of Type II error. Consequently, null findings should be interpreted with caution, as the study may not have been sufficiently powered to detect smaller, yet potentially meaningful, effects. Albeit BFRT is safe among PWH during a small number of sessions, specialists should carefully consider tailored prophylaxis for individual participation. The current findings reflect the acute responses of two sessions of BFRT. Future studies should evaluate the long-term adaptations to BFRT in PWH, as cardiovascular, neuromuscular, and perceptual effects may differ after repeated sessions due to adaptation, sensitization, or cumulative effects (e.g., joint stress in hemophilia).

## 5 Conclusions

AUTO and NAUTO conditions at 50% LOP appear to be equally tolerable and safe in severe PWH, under prophylaxis treatment, during an upper limb standard BFRT protocol, without great increases in RPE or pain. However AUTO showed trends for improved cardiovascular and neuromuscular responses. AUTO condition induced a hypotensive and hypoalgesic acute response, albeit no between-group differences were found. The AUTO condition resulted in increased muscular activation in the triceps brachii during the final cycles of the first sets, as well as higher VAS scores. The current findings reflect the acute responses to BFRT and long-term responses in PWH may differ due to adaptation, sensitization, or cumulative effects.

## Data Availability

The raw data supporting the conclusions of this article will be made available by the authors, without undue reservation.
